# Effectiveness of a multifaceted implementation strategy on physicians’ referral behavior to an evidence-based psychosocial intervention in dementia: a cluster randomized controlled trial

**DOI:** 10.1186/1471-2296-14-70

**Published:** 2013-05-30

**Authors:** Carola ME Döpp, Maud JL Graff, Steven Teerenstra, Maria WG Nijhuis-van der Sanden, Marcel GM Olde Rikkert, Myrra JFJ Vernooij-Dassen

**Affiliations:** 1 Radboud University Nijmegen Medical Centre, Scientific Institute for Quality of Healthcare (IQ healthcare), Nijmegen, the Netherlands; 2 Radboud Alzheimer Centre, Radboud University Nijmegen Medical Centre, Nijmegen, the Netherlands; 3 Department of Rehabilitation, Radboud University Nijmegen Medical Centre, Nijmegen, the Netherlands; 4 Department for Health Evidence (Biostatistics section), Nijmegen, the Netherlands; 5 Department of Geriatrics, Radboud University Nijmegen Medical Centre, Nijmegen, the Netherlands; 6 Department of Primary and Community Care, Radboud University Nijmegen Medical Centre, Nijmegen, the Netherlands; 7 Kalorama Foundation, Beek-Ubbergen, the Netherlands; 8 Scientific Institute for Quality of Healthcare (IQ healthcare), Radboud University Nijmegen Medical Centre, 114 IQ Healthcare, P.O. Box 9101, , Nijmegen, 6500 HB, the Netherlands

## Abstract

**Background:**

To evaluate the effectiveness of a multifaceted implementation strategy on physicians’ referral rate to and knowledge on the community occupational therapy in dementia program (COTiD program).

**Methods:**

A cluster randomized controlled trial with 28 experimental and 17 control clusters was conducted. Cluster included a minimum of one physician, one manager, and two occupational therapists. In the control group physicians and managers received no interventions and occupational therapists received a postgraduate course. In the experimental group physicians and managers had access to a website, received newsletters, and were approached by telephone. In addition, physicians were offered one outreach visit. In the experimental group occupational therapists received the postgraduate course, training days, outreach visits, regional meetings, and access to a reporting system. Main outcome measure was the number of COTiD referrals received by each cluster which was assessed at 6 and 12 months after the start of the intervention. Referrals were included from both participating physicians (enrolled in the study and received either the control or experimental intervention) and non-participating physicians (not enrolled but of whom referrals were received by participating occupational therapists). Mixed model analyses were used to analyze the data. All analyses were based on the principle of intention-to-treat.

**Results:**

At 12 months experimental clusters received significantly more referrals with an average of 5,24 referrals (SD 5,75) to the COTiD program compared to 2,07 referrals in the control group (SD 5,14). The effect size at 12 months was 0.58. Although no difference in referral rate was found for the physicians participating in the study, the number of referrals from non-participating physicians (t −2,55 / 43 / 0,02) differed significantly at 12 months.

**Conclusion:**

Passive dissemination strategies are less likely to result in changes in professional behavior. The amount of physicians exposed to active strategies was limited. In spite of this we found a significant difference in the number of referrals which was accounted for by more referrals of non-participating physicians in the experimental clusters. We hypothesize that the increase in referrals was caused by an increase in occupational therapists’ efforts to promote their services within their network.

**Trial registration:**

NCT01117285

## Background

In 2040 the number of people with dementia is expected to be 81.1 million worldwide [1]. In the Netherlands 70% of these people live in the community [2]. Several psychosocial interventions have proven to be effective in increasing the quality of life of people with dementia and / or their caregivers [3-12]. Implementation of these interventions is necessary to improve the quality of health care. Physicians have an important role in the implementation of these interventions as they serve as gatekeepers that provide people with access to other healthcare services. In spite of growing attention for implementation in the area of psychosocial interventions for people with dementia (e.g. [13,14]) no studies have evaluated the effect of implementation strategies on physicians’ referral behavior regarding psychosocial interventions for people with dementia living in the community.

The community occupational therapy in dementia (COTiD) program is an example of a psychosocial intervention. COTiD is a client-centered and family-based intervention that consists of 10 one-hour sessions in the clients’ home environment [15]. The intervention aims to increase or maintain functional independence, social participation, and quality of life of both the person with dementia and the caregiver [15]. The program was proven to be (cost) effective in improving the clients’ daily functioning and in improving the quality of life, general health, and mood of both the client and caregiver. In addition, a significant increase in caregivers’ sense of competence was found [5,6,16]. In spite of these positive effects, only 20% of the occupational therapists educated in using the program utilized it in clinical practice [17]. One of the main barriers was a lack of referrals due to insufficient knowledge of physicians about the COTiD program, not experiencing psychosocial interventions to be part of their frame of reference, and experiencing a lack of contact with occupational therapists in their network [17]. The lack of referrals and therewith the lack of experience resulted in a low feeling of competence of the occupational therapists [17]. These barriers were used as a basis to create an implementation strategy aimed to decrease these barriers and increase the utilization of the COTiD program in clinical practice.

Multifaceted implementation strategies are more likely to result in change in professional behavior compared to educational strategies [18-22]. As physicians, managers, and occupational therapists are responsible for care delivery according to the COTiD program we developed a multifaceted implementation strategy that targets these professionals. The overall aim of the strategy was to increase the number of referrals to this intervention and to increase occupational therapists adherence to the program. This paper reports on the results of a cluster randomized controlled trial regarding the effect of the multifaceted implementation strategy on the number of referrals of people with dementia to occupational therapy according to the COTiD program per cluster and on physicians’ knowledge of the COTiD program. Effect of the implementation strategy on occupational therapists’ knowledge and adherence, managers knowledge, client and caregiver treatment outcomes, and cost-effectiveness will be reported elsewhere. This article is written according to the latest CONSORT guidelines of randomized controlled trials.

## Methods

### Design and participants

A single blinded cluster randomized controlled study with 45 clusters was conducted between January 2009 and December 2011. A cluster was defined as a functional unit delivering outpatient occupational therapy services. The eligibility criteria for clusters was that for each cluster at least one physician, one manager, and two occupational therapists were able to participate in the study. In order to prevent contamination, each professional was only allowed to participate in one cluster. Clusters were recruited between January and October 2009 from hospitals, nursing homes, and mental health services that delivered community occupational therapy in one of three regions in the Netherlands (Nijmegen, Amsterdam, and Rotterdam). Occupational therapists were required to complete a post-graduate course on the COTiD program prior to the study. No specific type of physician was targeted, however we only included physicians who reported that they were able to include at least eight client-caregiver couples eligible for the COTiD program (people with mild to moderate dementia living at home and their caregiver). Finally, we only included managers that were responsible for directly or indirectly facilitating occupational therapy at home for people with dementia. Eligibility of clusters was checked by two research assistants. Eligible clusters were stratified by type of setting and randomly assigned to the control or experimental group in a 2:1 ratio by an independent statistician. This ratio was chosen because of data collection at client and caregiver level. It was expected that we needed twice as much control clusters to recruit a sufficient amount of client and caregiver couples in this group. The following criteria were used for the inclusion of client and caregiver couples: 1) the client needed to be diagnosed with mild or moderate dementia (MMSE 10 – 24), 2) the client was not diagnosed with depression or severe behavioral problems as judged by the referring physician, 3) the client needed to live in the community, and 4) the client had a caregiver that provided care at least twice a week. More comprehensive information on the methods used to evaluate client and caregiver outcomes are reported elsewhere [23]. All participants were requested to complete a consent form.

### Interventions

#### Control group

Physicians and managers did not receive any interventions. Occupational therapists received a 3-day post-graduate course, mainly consisting of lectures, discussions on the content of the COTiD program, and homework assignments including reading and one practical assignment.

#### Experimental group

The multifaceted implementation strategy targeted physicians, managers, and occupational therapists involved in the delivery of care to people with dementia and their caregiver living in the community. The complete strategy is described in a previous publication [23]. The role of the physicians is to refer eligible patients to the COTiD program for which awareness and knowledge is necessary. Managers regulate the supply and demand of occupational therapy care and need to facilitate this service. For this purpose sufficient knowledge on the COTiD program is necessary. To increase knowledge and awareness physicians and managers were provided with access to an educational website and were sent four newsletters. In addition, physicians were contacted by phone at least once and were offered an outreach visit in which the COTiD program was more thoroughly explained. As collaboration between professionals may enhance implementation [24], occupational therapists were offered two training days and five to seven outreach visits in which extensive time was spent on improving occupational therapists skills in promoting the COTiD program among physicians and their skills in working together with their network. All interventions were offered during a one-year period.

### Measurement instruments

#### Referral rate – primary outcome

Data on referrals were collected at cluster level. Occupational therapists sent depersonalized copies of all community occupational therapy referrals of people with dementia to the research team. Referrals were included from both participating and non-participating physicians. We defined participating physicians as those physicians that were enrolled in the study and received either the control or experimental intervention. Non-participating physicians were those physicians that were not enrolled but of whom referrals were received by the participating OTs. Referrals were included in the analysis if they referred to community occupational therapy and it concerned a person diagnosed with dementia. For each referral, information was collected on the date of birth, gender, diagnosis, and MMSE score of the client. In addition, data were collected on the date of referral, the referral question, and on the type of physician. During the study, period reminders were sent to all participating occupational therapists. Referrals were categorized as referring to the COTiD program or not referring to the COTiD program. Referrals to the COTiD program needed to specifically mention the program or needed to specify that therapy or advice was requested regarding daily activities in the home environment of the client and caregiver. Referrals that did not meet these criteria were categorized as “not referring to the COTiD program”. Referrals were categorized independently by two of the authors (CD, MG). One of the assessors (MG) was blinded for group allocation. Results were compared and discussed until 100% consensus was reached.

#### Knowledge of physicians on the COTiD program – secondary outcome

An electronic close-ended questionnaire was developed to assess physicians’ knowledge of the COTiD program. A personal link to the questionnaire was provided by email at baseline and at 6 and 12 months follow-up. The questionnaire consisted of eight questions. The first question included eight short case descriptions for which physicians needed to indicate if the clients in these descriptions were eligible for treatment according to the COTiD program. Additional questions related to physicians’ knowledge of the effectiveness, cost-effectiveness, and general content of the COTiD program. The remaining questions concerned the reimbursement of the COTiD program, facilitation of the program in clinical practice, and the effectiveness of pharmacological versus non-pharmacological interventions. Face validity was obtained during an expert panel meeting with expert occupational therapists. Higher knowledge scores indicate greater knowledge.

### Blinding

The study was single blinded: the research assistant who acquired the data (IM) was blinded for group allocation. It was not possible to blind professionals for group allocation.

### Sample size

An average of 30 patients per year per institute was expected to be available for referral to community occupational therapy services based on statements of physicians of different settings (25 patients per year in nursing homes, 35 patients per year in general hospitals, and 25 patients per year in mental health services). Furthermore, the availability of two occupational therapists per institute is reasonable. We decided to recruit 30 control clusters and 15 experimental clusters on the base of the following reasoning. Given an ICC of 0.20, the effective sample size per cluster is (cluster size)/design effect = 30/6.8 = 4.5. Thus the effective sample size of experimental clusters is 68 versus 135 in the control clusters. This produces a power of 97% to detect a difference of 0.25 versus 0.05 for the number of referrals.

### Data analysis

Baseline characteristic of occupational therapists, physicians, and managers between groups were compared using t-tests for parametric data and chi-square tests for non-parametric data. A two-sided significance level of 0.05 was used for all statistical tests.

#### Referral rate – primary outcome

Chi-square tests were executed to assess the difference between groups regarding the number of clusters that did not receive any referrals. Mixed model analysis was used to evaluate the difference between groups regarding the number of referrals to the COTiD program. The mixed model accounted for clustering of times of measurement and for the interaction between time of measurement and the type of implementation strategy. The effect size was calculated using Cohen's *d*. Covariate analyses using multilevel analyses were conducted to uncover factors that had the most influence on the number of COTiD referrals.

Based on the research teams’ expectations of their possible influence on the referral rate, six covariates were used for further analysis. As referrals were measured at the cluster level we were only able to include covariates that were also measured at the cluster level. Covariates were: the number of participating physicians, managers, and occupational therapists in each cluster, whether or not occupational therapists in one cluster worked at the same organization, the mean number of coaching sessions received by each cluster, and the type of organization. To prevent over fitting, the number of variables in the model needed to be limited to nine. Four variables were already included in the basic model to account for clustering of times of measurement and interaction between time of measurement and the type of implementation strategy. Therefore, we were only able to include an additional five (out of six) covariates. To select the model(s) with the best fit, 15 sets of eight or nine variables were prepared. The fit of these models was compared based on the information criteria (IC) (−2 log likelihood).

#### Physicians knowledge – secondary outcome

Differences between groups regarding physicians’ knowledge on the COTiD program were analyzed using mixed model analyses. Clustering of professionals and the interactions between time of measurement and type of implementation strategy were taken into account as fixed effects. Each question on the questionnaire was analyzed separately.

### Ethical approval

The research team submitted materials to the Human Subjects Committee of the region Nijmegen / Arnhem. This committee decided that further evaluation by the committee was not required as the data reported in this manuscript was collected from healthy healthcare professionals using low-burden questionnaires. Prior to data collection all participants were asked to complete a consent form.

## Results

### Baseline characteristics

**Figure 1:**
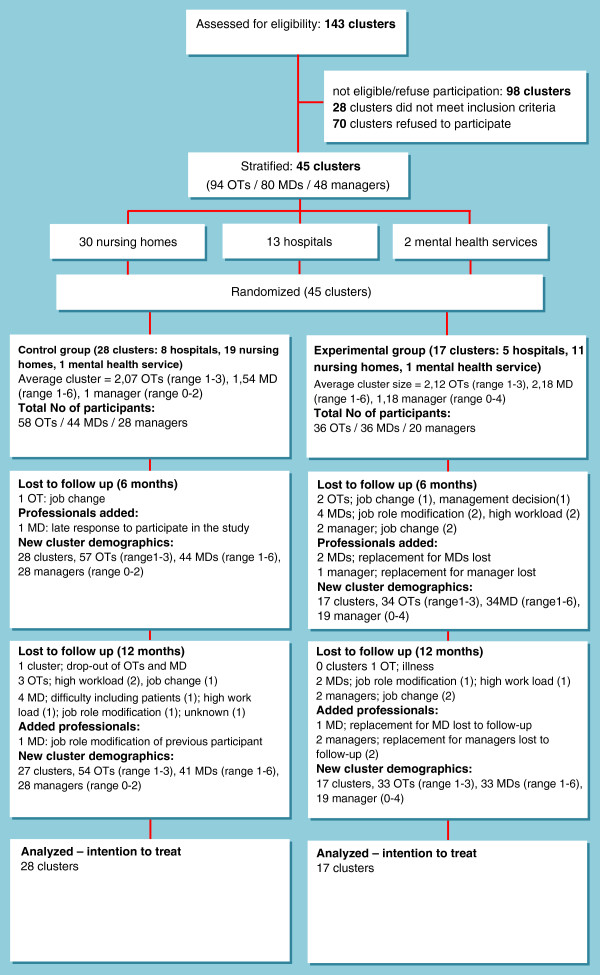
**Flow of participants through the trial. **OT = occupational therapist; MD = physician.

The required 45 clusters were recruited. A total of 80 physicians, 48 managers, and 94 occupational therapists participated at baseline. Cluster characteristics and the flow of participants through the trial are displayed in Figure 1. Baseline characteristics of physicians and occupational therapists (Table 1) showed no significant differences between groups. Physicians were either general practitioners or medical specialists. Medical specialists included geriatricians, neurologists, and nursing home physicians. At baseline only a significant difference was found in the average working experience of managers, which was twice as much in the control group (13,8 SD 8,96 versus 7,7 SD 3,8). Blinding was revealed for four clusters of which one control cluster and three experimental clusters.

**Table 1:** Baseline characteristics of participating physicians and occupational therapists

**Physicians**	**Experimental group**		**Control group**		**P-value**
Type of physician, N (%)					0,92
General practitioner	11 (30,6%)		13 (29,5%)		
Medical specialist	25 (69,4%)		31 (70,5%)		
Age, mean (SD)	49,7 (7,5)	(n = 29)	48,6 (8,0)	(n = 38)	0,60
Range	36-63		26-61		
Women, N (%)	17 (47,2%)		17 (38,6%)		0,44
Active as MD (years), mean (SD)	22,0 (7,1)	(n = 27)	20,74 (7,1)	(n = 35)	0,50
Range	10-34		6-35		
Experience dementia (years), mean (SD)	17,1 (6,6)	(n = 27)	17,2 (7,1)	(n = 33)	0,98
Range	5-30		5-31		
Specialization in Geriatrics, N (%)	17 (53,1%)	(n = 32)	24 (58,5%)	(n = 41)	0,64
Specialized (years), mean (SD)	11,8 (7,4)	(n = 15)	11,6 (7,7)	(n = 24)	0,94
Range	3-24		1-26		
**Occupational therapist (OT)**					
Age (years), mean (SD)	38,5 (10,7)		35,8 (9,9)		0,23
Range	22 – 58	(n = 36)	22 – 57	(n = 54)	
Women, N (%)	36 (100%)		54 (93,1%)		0,11
Qualified (years), mean (SD)	13,7 (8,9)	(n = 34)	13,4 (10,1)	(n = 44)	0,90
Active as OT (years), mean (SD)	13,7 (8,5)	(n = 35)	13,2 (9,4)	(n = 44)	0,96
Post-graduate completed (months), mean (SD)	13,0 (21,8)		13,7 (23,1)		0,90
Range	1 – 120	(n = 34)	1 – 101	(n = 43)	
Experience dementia (years), mean (SD)	7,5 (6,2)	(n = 35)	7,8 (7,1)	(n = 43)	0,81
Cases according to COTiD*, N (%)					0,25
0	8 (22,9%)		12 (27,3%)		
1-5	21 (60%)		29 (65,9%)		
6-10	3 (8,6%)		3 (6,8%)		
11-15	3 (8,6%)		0 (0%)		
16-20	0 (0%)		0 (0%)		
More than 20	0 (0%)		0 (0%)		

### Referral rate

During a one-year period 411 referrals of people with dementia to occupational therapy services were collected. Based on the eligibility criteria, 307 referrals were included in the analysis of which 111 of the control group and 196 of the experimental group. The number of referrals per cluster ranged from 0 to 17 in the control group and from 0 to 13 in the experimental group. Referrals were categorized independently by two researchers resulting in an inter-rater agreement of 94,5%. After discussion 100% consensus was reached.

#### Number of clusters receiving referrals

The number of clusters that did not receive any referrals to community occupational therapy services was significantly higher in the control group at both 6 months (χ^2^ 9,27; 1 ; p = .002) and 12 months (χ^2^ 9,94; 1; p = .002). At 12 months 16 (57,1%) of the control clusters still did not receive any community occupational therapy referrals compared to zero of the experimental clusters.

#### Number of COTiD referrals

At 6 months there was no significant difference between the number of COTiD referrals between groups (difference in change from baseline to 6 months: 1.2, 95%-CI from −1,42 to 3,90). However, at 12 months the mean number of COTiD referrals per cluster was significantly higher in the experimental group: difference in change from baseline to 12 months: 3.2, 95%-CI from 0.50 to 5.8 with an average of 2,07 referrals (SD 5,14) in the control group and 5,24 referrals (SD 5,75) in the experimental group. The effect size at 12 months was 0.58 which is considered a medium effect. Covariate analysis showed that none of the models was better than the basic model. This means that none of the covariates included had a significant influence on the number of COTiD referrals.

#### Participating versus non-participating physicians

As referrals were analyzed per cluster, referrals of both physicians participating in the study and those of non-participating physicians were collected. We therefore conducted an additional analysis to evaluate the difference between groups regarding the number of referrals of participating physicians and regarding the number of referrals from non-participating physicians. This analysis showed that there was no difference between groups in referrals from participating physicians at 12 months (t −1,27 / 43 / 0,21). However, there was a significant difference at 12 months regarding the number of referrals from non-participating physicians (t −2,55 / 43 / 0,02) with more referrals of non-participating physicians in the experimental group.

### Knowledge of the COTiD program

The response to the knowledge questionnaire was 52,5% (42/80) at baseline, 67,9% (53/78) at 6 months, and 59,5% (44/74) at 12 months. The number of non-responders was significantly higher in the control group at 6 months (χ^2^ 5,08; 1; p = .024). Overall knowledge of most subjects was moderate. Knowledge on the cost-effectiveness of the COTiD program was low in both groups (Table 2). No significant differences between groups were found regarding physicians’ knowledge on the COTiD program at 6 and 12 months follow-up (Table 3).

**Table 2:** Mean scores and group differences regarding physicians’ knowledge on the COTiD program

	**Score range**	**Baseline**			**6 months**			**12 months**		
		**Experimental mean (SD)**	**Control mean (SD)**	**Group diff**	**Experimental mean (SD)**	**Control mean (SD)**	**Group diff**	**Experimental mean (SD)**	**Control mean (SD)**	**Group diff**
1 – Eligibility of clients	0-8	5,65 (1,12)	5,92 (1,08)	−0,36	5,75 (1,21)	5,48 (1,33)	0,27	5,82 (1,43)	5,67 (1,33)	0,15
2 - Effect on client	0-9	4,35 (2,09)	4,52 (1,78)	−0,17	4,21 (2,04)	5,00 (1,29)	−0,79	4,65 (1,80)	4,96 (1,63)	−0,31
3 – Effect on caregiver	0-9	4,24 (1,95)	5,28 (2,05)	−1,04	4,50 (2,06)	5,48 (1,16)	0,98	5,18 (1,47)	5,56 (1,16)	−0,38
4 - General content	0-4	2,53 (0,87)	3,08 (0,86)	−0,55	0,0 (0,0)	0,0 (0,0)	0	3,18 (0,39)	2,89 (1,22)	0,29
5 - Pharmacological vs. non-pharmacological	0-4	2,24 (1,09)	2,00 (1,12)	0,24	1,89 (1,10)	2,12 (1,20)	−0,23	1,47 (1,07)	1,59 (0,84)	−0,12
6 – Facilitation	0-3	2,06 (0,90)	2,36 (0,70)	−0,3	1,86 (0,89)	1,96 (0,84)	−0,1	1,88 (0,93)	2,19 (0,96)	−0,31
7 – Cost-effect	0-3	0,0 (0,0)	0,04 (0,20)	−0,04	0,14 (0,36)	0,04 (0,20)	0,1	0,06 (0,24)	0,11 (0,32)	−0,05
8 – Reimbursement	0-5	3,88 (1,45)	3,54 (1,33)	0,34	4,39 (0,96)	3,08 (1,75)	1,31	4,12 (1,65)	3,63 (1,64)	0,49

**Table 3:** Results of the multivariate analyses regarding the difference in physicians’ knowledge per question

	**Difference between group in change from baseline to 6 months**		**Difference between groups in change from baseline to 12 months**	
	***Estimate***	***95% Confidence Interval***	***Estimate***	***95% Confidence Interval***
1 - Eligibility of clients	0,35	−0,37 to 1,08	0,40	−0,38 to 1,18
2 - Effect on client	−0,37	−1,25 to 0,52	−0,01	−0,98 to 0,97
3 - Effect on caregiver	−0,53	−1,69 to 0,63	−0,22	−1,09 to 0,65
4 - General content	−0,12	−1,42 to 1,18	−0,01	−1,01 to 0,98
5 - Pharmacological vs. non-pharmacological	−0,27	−0,79 to 0,26	−0,11	−0,64 to 0,42
6 - Facilitation	0,03	−0,42 to 0,48	−0,08	−0,53 to 0,37
7 - Cost-effect	0,05	−0,12 to 0,22	−0,01	−0,19 to 0,17
8 - Reimbursement	0,77	−0,21 to 1,75	0,10	−0,87 to 1,08

### Exposure of physicians to the multifaceted implementation strategy

A total of 11 physicians dropped out of the study (see Figure 1). Their replacements were requested to participate in the study. Those in the experimental group were provided with newsletters sent prior to their participation and with the link to the educational website.

More than half (67,5%) of the physicians in the experimental group were contacted by phone. However, almost a third of the physicians could not be reached (including physicians that dropped out) even after multiple attempts during a one-year period. The mean time spent on telephone contact with those physicians that could be reached was 15,15 minutes (SD 6,98). Six of the 36 physicians contacted by phone agreed to meet with the interventionist and the clusters’ occupational therapist to discuss the COTiD program in person. Additional data on exposure to the implementation strategy are displayed in Table 4. Analysis showed no relation between exposure to the different interventions and the number of COTiD referrals.

**Table 4:** Exposure of physicians in the experimental group to the multifaceted implementation strategy

	**Frequency**	**Percentage**	**N**	**Missing**
**Nr of telephone calls, M (SD)**	1,1 (0,93)		40	0
0 times, N (%)	13	32,5%		
1 time, N (%)	12	30%		
2 times, N (%)	13	32,5%		
3 times, N (%)	2	5%		
**Time per telephone call**	10,23 (9,17)		40	0
M (SD)				
**Visited website ≥ 1**	12	60%	20	20
N (%)				
**Read ≥ 1 newsletters**	23	92%	25	15
N (%)				
**Physicians visited by the interventionist**	6	15%	40	0
N (%)				

## Discussion

The results show that our experimental multifaceted implementation strategy is more effective in increasing the number of referrals to occupational therapy according to the COTiD program compared to the standard post-graduate course that only focused on occupational therapists. In spite of the large and increasing amount of community dwelling people with dementia cared for by informal caregivers, the number of referrals was still relatively low in both groups and needs further attention. No differences between groups were found regarding physicians knowledge of the COTiD program.

A review on outpatient referral behavior [25] and findings of the general implementation literature [20,26,27] stated that passive dissemination strategies are less likely to result in changes in professional behavior. Although we offered both passive and active strategies only a limited amount of physicians was exposed to the active strategies. In spite of this, we found a significant difference in the number of referrals. This may be explained by the subgroup analysis that showed that this significant increase in the number of referrals was not the effect of the interventions aimed at the participating physicians but was fully accounted for by more referrals of the non-participating physicians in the experimental clusters compared to the non-participating physicians in the control clusters. Also, we did not find a significant difference between groups regarding physicians’ knowledge which was only moderate in both groups. We hypothesize that our efforts to increase occupational therapists’ skills to promote community occupational therapy services were the effective component of the experimental strategy. Although we did not record the actions undertaken by the occupational therapists to promote the COTiD program, it is likely that occupational therapists in the experimental group put more effort into promoting occupational therapy within their network. Zwarenstein et al. (2009) report that better and more intensive inter-professional collaboration may positively affect healthcare outcomes [24]. In other words, further improvement of the collaboration between physicians and occupational therapists may lead to an increase in the amount and appropriateness of referrals and therewith clients’ access to community occupational therapy services.

### Strengths and limitations

In spite of the importance of referral behavior for implementation of effective interventions only few studies evaluated the effect of implementation strategies on physicians’ outpatient referral behavior [25]. Our study contributes to this limited knowledge on effective strategies to change referral behavior. As we evaluated a multifaceted strategy and due to the chosen study design we cannot state with certainty which component(s) of the strategy caused the increased referral rate in the experimental group. Further process analysis is recommended to explain the study results by evaluating physicians, managers, and occupational therapists’ experiences.

The lack of effect regarding physicians’ knowledge could be the result of our recruitment method. To convince physicians to participate in the study we used publications on the effect of the COTiD program. In addition, this recruitment method may have led to a limited feeling of necessity to receive additional information through the website, newsletters, telephone calls and outreach visits. Last, the difficulty to reach physicians suggests that barriers exist that relate to the attitude of the physician regarding psychosocial interventions such as the COTiD program and / or practical barriers such as workload pressure.

For data collection on the number of referrals we relied on the participating occupational therapists. Therefore it is likely that we missed data. However, to decrease the amount of missing data we sent occupational therapists of both groups several reminders. Physicians’ knowledge on the COTiD program was based on a close-ended questionnaire. Face validity was established using an expert panel, but reliability of the questionnaire was not assessed. At six months there was a significant difference in the response rate to this knowledge questionnaire which may have caused bias. During the study several physicians changed jobs or their role within the organization changed. Although their replacements received access to the website and were provided with the previously sent newsletters, they had less time to change their referral behavior. However, these situations occur in daily practice and the results therefore show the actual benefit of the implementation strategy in clinical practice. Patient characteristics may have influenced whether or not physicians referred people to occupational therapy, however our analysis did not allow to correct for these type of characteristics as they were not at the cluster level.

The multifaceted implementation strategy aimed to stimulate occupational therapists to promote the COTiD program. Although this may have contributed to the increased number of referrals a more direct approach to stimulate collaboration may result in even better outcomes. Several studies showed that including an inter-professional training component was successful in improving care (e.g. [28]) or in improving inter-professional attitudes and self-reported team skills [29].

## Conclusion and implications

Psychosocial interventions have shown to have positive effects and the use of these interventions in dementia care are included in European dementia guidelines [30] including two Dutch guidelines [31,32]. Physicians serve as gatekeepers and are in the position to provide clients and caregivers with access to psychosocial services using referrals. Increasing the number of referrals to evidence-based psychosocial interventions is a first step to implementation. Our study showed that the number of referrals can be improved using a multi-professional approach. The results suggest that the use of passive dissemination strategies such as websites and newsletters were not effective, but that encouraging occupational therapists to promote their services within their network did contribute to the increased number of referrals. Establishing close inter-professional collaboration within the professionals’ network may further increase the number of referrals. We therefore encourage physicians and clinicians providing psychosocial interventions to more actively collaborate in order to gain a better understanding of each other’s services and improve clients’ access to care. Healthcare managers have an important task in facilitating this collaboration.

As there are still a limited number of studies, future studies to implementation of evidence-based psychosocial interventions should include referral behavior as an outcome measure. Although we only included the number of referrals the quality of referrals is an important aspect as well that should be considered in future research. As different interventions and professionals come with different barriers the degree to which the results of the study can be generalized is limited and implementation strategies should always be adapted to barriers experienced by the specific target group.

## Competing interest

The authors declare that they have no competing interests.

## Authors’ contributions

CD was the investigator and supervised the execution of the implementation strategy, carried out data collection, the analysis, and wrote the paper. MG, MVD, RN, ST, and MOR were responsible for the design, project supervision, and writing. MG and ST contributed to data analysis. MG and MVD were the guarantors. All authors read and approved the final manuscript.

## Data sharing statement

Complete data sets of all reported outcomes can be provided on request of fellow researchers.

## Pre-publication history

The pre-publication history for this paper can be accessed here:

http://www.biomedcentral.com/1471-2296/14/70/prepub
